# The Association Between Shared Decision-Making and Child Health in the General Pediatric Population

**DOI:** 10.1007/s10995-026-04251-6

**Published:** 2026-04-15

**Authors:** Alyssa Cohen, Kelly N. Michelson, Michelle L. Macy, Nia Heard-Garris, Kristin Kan

**Affiliations:** 1 Division of Advanced General Pediatrics and Primary Care, Ann & Robert H. Lurie Children’s Hospital of Chicago, 225 E. Chicago Ave, Box 162, Chicago, IL 60611 USA; 2 Department of Pediatrics, Northwestern University Feinberg School of Medicine, Chicago, IL USA; 3 Mary Ann & J. Milburn Smith Child Health Outcomes, Research and Evaluation Center, Stanley Manne Children’s Research Institute, Ann & Robert H. Lurie Children’s Hospital of Chicago, Chicago, IL USA; 4 Division of Critical Care Medicine, Ann & Robert H. Lurie Children’s Hospital of Chicago, Chicago, IL USA; 5 Division of Emergency Medicine, Ann & Robert H. Lurie Children’s Hospital of Chicago, Chicago, IL USA

**Keywords:** Pediatrics, Shared decision-making, Patient-provider communication, Health services research

## Abstract

**Objective:**

Shared decision-making (SDM) is a decision-making approach that aims to enhance collaboration between patients and providers. SDM is associated with improved health outcomes and decreased healthcare expenditure among children with special health care needs. However, much remains unknown about the role of SDM in the broader general pediatric population. We sought to describe associations between SDM and child health and healthcare utilization in a national pediatric sample.

**Methods:**

Using data from the 2019–2021 National Survey of Children’s Health, we examined the association between SDM and forgone health care, missed school days due to illness or injury, and emergency department (ED) visits. Bivariate and multivariable logistic regression analyses were performed and adjusted for sociodemographic variables.

**Results:**

The study sample included 31,791 children who needed medical decisions made in the prior 12 months (mean age 8.7 years, 42.3% with special health care needs). Most (85%) experienced SDM. In adjusted analysis, SDM was associated with significantly lower odds of children forgoing needed health care (adjusted odds ratio [aOR] = 0.22, 95% confidence interval [CI] 0.17–0.27), and lower odds of > 4 missed school days (aOR = 0.80, 95% CI 0.66–0.95). Experiencing SDM was not associated with ED visits.

**Conclusions for Practice:**

SDM may confer benefits for all children in a general pediatric population. Experiencing SDM was associated with less forgone health care and fewer missed school days due to illness or injury. Future work should explore approaches to SDM that consider the needs and preferences of families across the spectrum of pediatric care.

**Supplementary Information:**

The online version contains supplementary material available at 10.1007/s10995-026-04251-6.

## Significance

Shared decision-making (SDM) is a key component of patient-centered care and associated with improved health and healthcare utilization outcomes for children with special health care needs. However, SDM is understudied in the broader general pediatric setting. This analysis of recent national data demonstrates that shared decision-making is associated with less forgone health care and fewer missed school days in a general pediatric population.

**Supplementary Information:**

The online version contains supplementary material available at 10.1007/s10995-026-04251-6.

## Introduction

Shared decision-making (SDM) is a key component of patient-centered communication in instances where multiple reasonable options exist for a clinical decision (Opel, 2018). SDM aims to build collaborative relationships between patients and providers through a bidirectional exchange of information, consensus building, and mutual agreement on a care plan to align with families’ values and goals (Charles et al., 1997) Committee On Hospital et al., (2012). SDM is also situated within the medical home model, as it aligns in emphasis on family engagement and effective care delivery (Lichstein et al., 2018). The use of SDM has been widely studied among children with special health care needs (CSHCN) with some encouraging results for improving chronic disease knowledge and outcomes (Brinkman et al., 2013; Fiks et al., 2015; Liu et al., 2018; Wyatt et al., 2015), increasing access to needed health services (Perez Jolles et al., 2020), and decreasing healthcare expenditure (Fiks et al., 2012). However, few studies have examined SDM in the general pediatric population, defined here as including all children with and without special health care needs. Attention to the impact of SDM in the general pediatric population may reveal cross-cutting opportunities for the application of SDM in multiple care settings (Wyatt et al., 2015).

Important health decisions occur in the general pediatric setting for all children and many of these decisions represent opportunities for SDM. In a 2018 framework for SDM in pediatrics, Opel cites several examples of decisions relevant to the general care of children that could benefit from SDM, including management of mild seasonal allergies, choices about male newborn circumcision, and decisions around infant breastfeeding (Opel, 2018). Prior studies demonstrate lower rates of reported SDM among certain populations, including children with emotional, developmental, or behavioral health conditions and children with multiple adverse childhood experiences (ACEs) (Chiam et al., 2021; Schweer-Collins and Lanier, 2021). In addition to experiencing lower rates of SDM, CSHCN from Black and Hispanic families also experience less benefit of SDM on healthcare utilization outcomes (Perez Jolles et al., 2020). Taken together, the extant literature points toward an opportunity to improve understanding and application of SDM across the entire general pediatric population.

Despite its recognition as a foundational pillar of high-quality pediatric care in multiple models, the impact of SDM on healthcare outcomes in the broader pediatric population – including children without special health care needs – remains understudied. The current analysis aims to describe the associations between SDM and child health and healthcare utilization in a nationally representative sample of children. We hypothesized that families experiencing SDM are more likely to report better child health, as indicated by report of fewer missed school days due to illness or injury. Additionally, families experiencing SDM will have more effective healthcare utilization, including less forgone health care and fewer emergency department (ED) visits. Better understanding of SDM may contribute new insight about improving health care communication for general pediatric patients and families and support optimal care models to improve health outcomes.

## Methods

### Data Source

This study used publicly available data from the 2019–2021 National Survey of Children’s Health (NSCH), an annual cross-sectional survey administered via web or paper by the U.S. Health Resources and Services Administration (HRSA) to a nationally representative sample of parents or caregivers (hereafter referred to as parents) of noninstitutionalized children in the United States (US). A total of 123,102 households completed surveys with response rates ranging from 40.3% to 42.4% across years. Each respondent completed the survey pertaining to one child living in the household. Detailed survey design and methodology are located in US Census publications (2019 National Survey of Children’s Health Methodology Report, (2020); 2020 National Survey of Children’s Health Methodology Report, (2021); 2021 National Survey of Children’s Health Methodology Report, (2022)).

### Study Sample

We included responses from parents who indicated “yes” to the question “During the past 12 months, did this child need any decisions to be made regarding his or her health care, such as whether to get prescriptions, referrals, or procedures?” (N = 31,791, 25.8%). Respondents indicating no health care visits or no medical decisions during the past 12 months (N = 89,968, 73.1%) were excluded from analysis, as were those with missing data for all SDM items (N = 1,343, 1.1%).

### Dependent Variables

We identified three outcomes of interest relevant to child health and healthcare utilization for a general pediatric population.

#### Forgone Health Care

Forgone health care was assessed with the yes/no question, “during the past 12 months, was there any time when this child needed health care, but it was not received?” (Wu and Brannon, 2023).

#### Missed School Days Due to Illness or Injury

 Survey questions about missed school days due to illness or injury were collected pertaining to children ages 6–17 years. Parents were asked to estimate the number of school days missed in the preceding 12 months due to illness or injury, including missed days of formal home schooling. From five categorical options in the NSCH, responses were dichotomized as “fewer than 4 days” versus “four or more days” missed. This cut point was selected based on distribution of responses with significant right-skew; the majority of children missed fewer than 4 days of school in the preceding year.

#### Emergency Department Visits

 ED visits were measured over the preceding 12 months as none, 1, or 2 or more. These responses were initially dichotomized as any ED visits (none versus 1 or more) and, due to significant right-skewed distribution, further analysis was performed to assess for differences between respondents indicating 1 ED visit and those with 2 or more visits.

### Independent Variables

The primary independent variable in this analysis was SDM, which was assessed as a composite measure of three survey items asking parents who indicated needing medical decisions in the preceding 12 months how often their child’s doctors or other health care providers: (1) “discuss[ed] with [parent] the range of options to consider for [child]’s health care or treatment, (2) “made it easy for [parent] to raise concerns or disagree with recommendations for [child]’s health care”, and (3) “worked with [parent] to decide together which health care and treatment choices would be best for the child” in the preceding 12 months. These questions are aligned with Charles’s landmark definition of SDM (1997) and information on survey item development can be found in prior publications measuring SDM in the National Survey of Children with Special Health Care Needs (NS-CSHCN) (Butler et al., 2015; Smalley et al., 2014). Consistent with these and other NS-CSHCN and NSCH publications, our composite SDM outcome measure was coded “yes” for responses of “usually” or “always” to all completed questions and coded “no” if the response was “never” or “sometimes” to any of the three components.

Covariates were selected based on prior literature linking sociodemographic and clinical factors to child health and healthcare access, as well as evidence of disparities in the use of SDM across many of these categories in pediatric populations (Flores 2010; Flores et al., 2017; Flores and Tomany-Korman, 2008; Ghandour et al., 2022). Covariates included child’s age, child’s sex, parent-reported ethnicity (i.e.., Hispanic or non-Hispanic) and race (i.e., white, Black, multi-racial, other [including American Indian or Alaska Native, Asian Indian, Chinese, Filipino, Japanese, Korean, Vietnamese, other Asian, Native Hawaiian, Guamanian or Chamorro, Samoan, other Pacific Islander]), health insurance type (i.e., uninsured, only public insurance, or any private insurance) and special health care needs status (yes or no, based on the five-question CSHCN screener) (*2020–2021 National Survey of Children’s Health (Combined 2-year dataset) STATA codebook for data users*, 2023. Household-level covariates included primary language (i.e., English or non-English) and parental education (i.e., less than high school degree, high school degree/GED, some college or technical school, or college degree or higher). Parental education was selected as the primary socioeconomic status indicator due to moderate collinearity with household income variables in this dataset and its conceptual relevance to communication and decision-making practices. We recognize that race is a social construct and racism is associated with significant health outcomes (Slopen et al., 2024). Thus, race and ethnicity were included as covariates because of well-documented pediatric health and healthcare disparities based on reported race and ethnicity, likely driven by racism, including differences in use of SDM (Flores, 2010; Heck and Parker 2002; Perez Jolles et al., 2020; Slopen et al., 2024; Trent et al., 2019).

### Statistical Analysis

Descriptive statistics summarized sociodemographic and child health measures across SDM categories, and chi-squared analyses assessed for differences between those with and without SDM.

The associations between SDM and outcome variables were modeled using unadjusted and adjusted multivariable logistic regression. Because the extant literature demonstrates robust associations between SDM and health and health care measures among CSHCN, sensitivity analyses were planned a priori to assess whether the responses from the subpopulation of CSHCN within our general pediatric sample were driving any observed associations between SDM and our key outcomes. Sensitivity analyses were performed among subpopulations of CSHCN and children without special health care needs, using adjusted multivariable logistic regression. Results from subpopulation analyses were confirmed with regression models that included an interaction term for SDM and special health care needs status, allowing for margins analyses to assess for effect modification. Analyses utilized survey sampling weights provided by the NSCH and survey commands to permit nationally representative inferences. A significance level of p ≤ 0.05 was used for all analyses. Statistical analyses were conducted with Stata version 19.5 software (StataCorp LLC, College Station, TX).

## Results

Of 123,102 total survey respondents, 31,791 were included in the analytic sample (Table 1). Mean age of included children was 8.7 years (standard deviation (SD) 5.3 years). Among the survey-weighted sample, 57.7% did not have special health care needs. Most respondents (85.0%) reported experiencing SDM with their child’s health care providers, with 15% not experiencing SDM based on survey responses. Associations were identified between SDM and sociodemographic variables, with less experience of SDM reported among families identifying as Hispanic, non-Hispanic Black, or non-Hispanic multiracial or other race, households speaking languages other than English, families with public insurance or no health insurance, and families with lower parental education. Families of CSHCN were less likely to report experiencing SDM than those without special health care needs in unadjusted analysis (82% vs 87%, p < 0.001) (Table 1).

**Table 1:** Sample demographic characteristics and child health care and utilization outcomes by shared decision-making (SDM) (N = 31,791 unless otherwise specified)

	Experienced SDM (n = 27,682) N (weighted %)^b^	Did not experience SDM (n = 4,109) N (weighted %)	*p*-value^a^
Age, mean (SD)	8.8 (5.4)	8.7 (4.9)	0.66
Child’s sex			0.67
Male	14,446 (85.2)	2,151 (14.8)	
Female	13,236 (84.8)	1,958 (15.2)	
Child’s ethnicity and race			< 0.001*
Hispanic	2,764 (76.3)	650 (23.7)	
Black, non-Hispanic	1,310 (83.4)	269 (16.6)	
Multiracial or other race, non-Hispanic	3,053 (82.8)	528 (17.2)	
White, non-Hispanic	20,555 (88.6)	2,662 (11.4)	
Parent education			< 0.001*
Less than high school degree	252 (66.5)	125 (33.5)	
High school degree or GED	2,291 (79.5)	479 (20.5)	
Some college or technical school	5,289 (81.4)	988 (18.6)	
College degree or higher	19,850 (88.6)	2,517 (11.4)	
Child with special health care needs			< 0.001*
No	15,106 (87.2)	1,888 (12.8)	
Yes	12,576 (82.0)	2,221(18.0)	
Household Language^c^			< 0.001*
English	26,744 (86.3)	3,753 (13.7)	
Non-English	847 (67.6)	338 (32.4)	
Insurance Type^d^			< 0.001*
Any Private Insurance	21,811 (87.4)	2,746 (12.6)	
Only Public Insurance	5,047 (80.2)	1,153 (19.8)	
Uninsured	605 (74.8)	167 (25.2)	

^a^*p-*value reflects Chi^2^ analyses (for categorical variables) and unadjusted linear regression (for continuous variables) comparing variables across shared decision-making responses

^b^All proportions are displayed as survey sample weighted percentages. Sample numbers displayed are unweighted

^c^N = 31,682

^d^N = 31,529

^*^Significance level p < 0.05

Experience of SDM was significantly associated with each outcome measure in unadjusted analysis (Table 2). This unadjusted analysis showed that children experiencing SDM were less likely to forgo needed health care (odds ratio [OR]: 0.20, 95%CI 0.15–0.25), to have four or more missed school days due to illness or injury (OR: 0.74, 95%CI 0.62–0.89), or to have or any ED visits (OR:0.75, 95%CI 0.63–0.90) than those who did not experience SDM.

**Table 2:** Unadjusted logistic regressions of shared decision-making (SDM) on child health and healthcare utilization outcomes

	Forgone health care		Missed school days		Any ED visit		Multiple ED visits	
	OR^a^	95% CI^b^	OR	95% CI	OR	95% CI	OR	95% CI
Experienced SDM?								
No	Reference		Reference		Reference		Reference	
Yes	0.20	0.15–0.25*	0.74	0.62–0.89*	0.75	0.63–0.90*	0.83	0.62–1.10

^a^*OR* odds ratio, ^b^*CI* confidence interval

^*^Significance level p < 0.05

All regressions utilize survey sample weighting for nationally representative inferences

In adjusted logistic regression, after controlling for covariates including special health care needs status, experiencing SDM was associated with 78% reduced odds of forgoing needed medical care (aOR: 0.22, 95%CI 0.17–0.27) compared to those not experiencing SDM (Table 3). SDM was also significantly associated with 20% decreased odds of missing 4 or more school days due to illness or injury (aOR: 0.80, 95%CI 0.66–0.95). The associations between SDM and any ED visits (0 vs 1 or more) and multiple ED visits (1 vs 2 or more) were not statistically significant in adjusted analysis. The regression analysis demonstrated significant associations between numerous covariates and each of the outcomes of interest, as noted in Table 3.

**Table 3:** Adjusted logistic regressions of shared decision-making (SDM) on child health and healthcare utilization outcomes

	Forgone health care		Missed school days		Any ED visit		Multiple ED visits	
	aOR^a^	95% CI^b^	aOR	95% CI	aOR	95% CI	aOR	95% CI
Experienced SDM?								
No	Reference		Reference		Reference		Reference	
Yes	0.22	0.17–0.27*	0.80	0.66–0.95*	0.92	0.76–1.11	0.97	0.72–1.31
Child’s age								
(continuous)	1.04	1.02–1.06*	1.01	0.99–1.02	0.97	0.96–0.98*	0.98	0.96–1.00
Child’s ethnicity and race								
White, non-Hispanic^c^	Reference		Reference		Reference		Reference	
Hispanic	1.14	0.81–1.61	1.04	0.84–1.29	1.28	1.04–1.58*	1.02	0.70–1.47
Black, non-Hispanic	0.94	0.64–1.38	0.57	0.46–0.70*	1.28	1.06–1.55*	1.21	0.86–1.69
Multiracial or Other	0.95	0.68–1.34	0.92	0.74–1.14	0.99	0.82–1.20	0.94	0.66–1.34
Parent education								
Less than high school degree	Reference		Reference		Reference		Reference	
High school degree or GED	0.91	0.46–1.81	1.04	0.63–1.72	0.81	0.52–1.26	0.59	0.33–1.04
Some college or technical school	1.12	0.58–2.15	0.75	0.46–1.22	0.76	0.50–1.17	0.54	0.31–0.92*
College degree or higher	0.72	0.37–1.43	0.59	0.36–0.95*	0.53	0.34–0.81*	0.43	0.25–0.74*
Child with special health care needs?								
No	Reference		Reference		Reference		Reference	
Yes	2.05	1.62–2.58*	1.68	1.49–1.90*	1.31	1.16–1.49*	2.33	1.81–2.99*
Household language								
English	Reference		Reference		Reference		Reference	
Non-english	1.04	0.63–1.72	0.55	0.37–0.80*	0.76	0.54–1.04	1.27	0.77–2.08
Insurance type								
Any private	Reference		Reference		Reference		Reference	
Only public	1.14	0.82–1.60	1.31	1.12–1.56*	1.61	1.37–1.88*	1.24	0.94–1.63
Uninsured	3.63	2.12–6.21*	0.89	0.57–1.38	1.79	1.22–2.62*	1.04	0.58–1.86

^a^aOR = adjusted odds ratio, ^b^CI = confidence interval

^c^Utilized as reference group because of previous literature demonstrating higher rates of SDM among non-Hispanic white families compared with other racial and ethnic groups

*Significance level p<0.05

All regressions utilize survey sample weighting for nationally representative inferences

In a stratified sensitivity analyses, both subpopulations with and without special health care needs who experienced SDM had significantly decreased odds of forgone needed health care (aOR: 0.18 and 0.29 respectively, 95% CI 0.14–0.24 and 0.19–0.44; Appendices 1–2). This was confirmed with a separate effect modification regression model, in which the term for interaction between SDM and CSHCN was not statistically significant (Appendix 3). For the outcome of missed school days due to illness or injury, among children without special health care needs, the outcome was not significantly associated with SDM (aOR: 0.98, 95%CI 0.74–1.29). In the effect modification regression model for missed school days, the interaction term between SDM and CSHCN was statistically significant (aOR: 0.69, 95% CI 0.48–0.99, Appendix 3). SDM was not significantly associated with ED visits in either subpopulation. Full results from the sensitivity analyses, including predicted probabilities across all SDM-CSHCN combinations, can be found in Appendices 1–4.

## Discussion

A collaborative approach to clinical communication and decision-making is considered standard of care for all children. Using a recent national dataset, we found that families of children in a general pediatric population who reported experiencing SDM with their child’s health care providers also reported fewer missed school days due to illness or injury and less forgone health care. While the impact of SDM on children with physical and mental health conditions has been well-described (Fiks et al., 2015; Lipstein et al., 2016; Perez Jolles et al., 2020; Smalley et al., 2014), this study is among the first to broaden the lens of SDM research to a more complete sample of children, which includes those with and without special health care needs. By situating SDM in an expanded clinical context, our results contribute evidence that high-quality communication is associated with improved health care access and outcomes across the spectrum of pediatric care decisions, while also highlighting areas for ongoing improvement that could benefit the pediatric population at large.

The landscape of medical decisions considered appropriate for SDM includes common decisions facing many families in a general pediatric setting, such as those surrounding preventive and routine healthcare. However, most literature to date has focused on CSHCN. CSHCN represent about 20% of US children (*Children and Youth with Special Health Care Needs NSCH Data Brief*, 2022, who have higher health care demands and utilization and therefore may face more medical decisions (Fiks et al., 2012). The existing body of evidence demonstrates a robust positive effect of SDM on health care outcomes among CSHCN prompting professional societies to call for its application in the care of this population (Committee On Hospital et al., (2012). More recent evidence points toward differences in health care and communication that may impact the health of other groups of children, such as those with exposure to ACEs, whose experiences may not be captured by studies focused solely on CSHCN (Schweer-Collins and Lanier, 2021). Our sensitivity analyses explored subpopulations of children with and without special health care needs and interactions between SDM and CSHCN status, with differential results in the strength and statistical significance of associations for our outcomes of interest. These findings underscore that SDM can be viewed as a foundational element of communication for all pediatric healthcare but may function differently among children with and without special health care needs.

We selected outcome variables for this analysis based on relevance to the health care of a general pediatric population. Forgone health care is a widespread issue affecting the health of millions in the pediatric population, and can stem from complex and interrelated barriers including structural, economic, and personal factors (Lichstein and Ghandour, 2020). Many of these barriers were amplified during the COVID-19 pandemic, which is likely captured in these data collected from2019 to 2021 (Gonzalez et al., 2021. In our analysis, families who reported receiving SDM had significantly lower odds of forgoing needed health care for their child, regardless of the child’s special health care needs status, after adjusting for sociodemographic characteristics. We hypothesize that the association between SDM and forgone care in the general pediatric population is twofold; first, parental report of receiving SDM may be more broadly indicative of a high-functioning system of care, such as a patient-centered medical home, in which barriers to access are more thoroughly addressed. Second, SDM may directly modify families’ care-seeking behavior by improving communication and trust with clinical teams. This association may also operate bidirectionally; families experiencing forgone healthcare may be less likely to engage in SDM practices. As such, SDM should be considered as a target factor for interventions to reduce forgone health care among children and adolescents.

Similarly, missed school days due to illness or injury has been used as a patient-reported indicator of child health in the context of medical home and quality of care assessment (Stevens et al., 2011). We hypothesized that high-quality SDM would decrease missed school days in a general pediatric population by ensuring families’ understanding of care plans and appropriate anticipatory guidance. Our results demonstrate a significant association between parental report of SDM and fewer missed school days in the overall population and among CSHCN; however, in the subpopulation of children without special health care needs, this association was no longer statistically significant. Sensitivity analyses demonstrate statistically significant effect modification of the association between SDM and missed school days by special health care needs status. This suggests that missed school days due to illness or injury among children without special health care needs are driven by factors other than SDM (e.g. acute or self-limited illnesses that do not require medical decisions to be made, school and childcare policies). Additionally, the statistical difference may reflect a floor effect given the overall lower rate of missed school days among children without special health care needs, making any incremental effect of SDM more challenging to detect (Allison et al., 2019; Forrest et al., 2011; Reuben and Pastor, 2013).

Our findings are consistent with extant literature in demonstrating significant associations between sociodemographic variables and SDM as well as our outcome variables (Ghandour et al., 2022). In the analyzed sample, experience of SDM was significantly less reported among families identifying as Hispanic, non-Hispanic Black, or non-Hispanic multiracial or other race, households speaking languages other than English, parents with less formal education, and those with publicly insured and uninsured children. These disparities may reflect not only differences in health care system access, but also in patient-provider communication. Prior evidence demonstrates that clinical communication can be directly affected by racial and language concordance between patients and providers (Ngo-Metzger et al., 2007; Shen et al., 2018); however, cultural background may also impact shared decision-making through different preferences for communication style and levels of involvement (Hawley and Morris 2017). This suggests that strategies for more equitable healthcare practices, and potentially more equitable health outcomes, will require a nuanced approach to application of SDM. Interventions should ensure that SDM is being responsively applied in pediatric populations, while preserving equity and autonomy (Perez Jolles et al., 2019, 2020).

While a key strength of this analysis is the use of a recent, large, nationally representative data set, important limitations are notable. The secondary, cross-sectional study design does not allow causal relationships to be ascertained. The primary predictor in this analysis, SDM, was measured based on parent report and is subject to recall and social desirability biases (Krumpal, 2013). Parents may or may not have recognized when clinical decisions were being made, and there are not details available on the types of decisions for which SDM was or was not applied. In addition, while items assessing SDM in this study assume that SDM includes joint parental and provider involvement in decision-making, this may not fully capture the breadth of SDM practices. As noted in prior studies, a sizeable number of parents may prefer a less active role in decision-making, particularly in clinically uncertain situations (Madrigal et al., 2012; Miller and Nelson, 2012; Tom et al., 2017). Many models of SDM acknowledge a continuum of practices that may constitute shared decision-making, reflective of this range of patient or parent preferences (Feudtner et al., 2018; Madrigal et al., 2012; Makoul and Clayman, 2006; Opel, 2018). It was not clear from our data whether parental involvement in decision-making was preferred by responding parents, or appropriate for the decision at hand, including in instances where it did not occur. Next steps in inquiry should include more nuanced assessment of decision-making and communication preferences among families in the general pediatric setting. It is important to consider the time period of the current analysis that includes survey years before and during the COVID-19 pandemic, which impacted healthcare utilization for children as previously noted (Wu and Brannon, 2023). Finally, for older children and adolescents who are gaining autonomy in self-management of their health and may interact with providers privately, parent report may not accurately reflect all experiences of health communication and decision-making. Incorporating youth-reported SDM measures and triangulating perspectives from parents, youth, and clinicians will be an important direction for future work.

## Conclusions

The current study demonstrates health and health care benefits of SDM for children in a general pediatric population, which includes those with and without special health care needs. Our findings support a role for SDM as an overarching theme of care in general pediatric settings with opportunities for improved clinical application. Future inquiry should continue to explore avenues to improve collaboration in health decision-making for children, incorporating provider, parent, and youth perspectives on SDM. Interventions to improve SDM should involve tailored approaches that take into consideration the needs and preferences of families across the spectrum of pediatric care.

## Supplementary Information

Below is the link to the electronic supplementary material.

Supplementary file1 (DOCX 33 KB)

## Data Availability

This study utilized publicly available datasets from the U.S. Census Bureau.

## Code Availability

Upon request.
