# Supplementary material for: The Association Between Shared Decision-Making and Child Health in the General Pediatric Population
Source: Matern Child Health J. 2026 Apr 15;30(4):575–83. doi: 10.1007/s10995-026-04251-6 (PMC13135518; doi:10.1007/s10995-026-04251-6)
Supplement: Supplementary file 1 — Supplementary file1 (DOCX 33 KB) [file 10995_2026_4251_MOESM1_ESM.docx]

| **Appendix 1: Adjusted Logistic Regressions of Shared Decision-Making (SDM) on Child Health and Healthcare Utilization Outcomes among Children WITH Special Health Care Needs subpopulation** | | | | | | | | | | | |
| --- | --- | --- | --- | --- | --- | --- | --- | --- | --- | --- | --- |
|  | **Forgone Health Care** | |  | **Missed School Days** | |  | **Any ED Visit** | |  | **Multiple ED Visits** | |
|  | **aOR**^a^ | **95% CI**^b^ |  | **aOR** | **95% CI** |  | **aOR** | **95% CI** |  | **aOR** | **95% CI** |
| **Experienced SDM?** | | |  |  | |  |  | |  |  | |
| No | *reference* | |  | *reference* | |  | *reference* | |  | *reference* | |
| Yes | 0.18 | 0.14-0.24* |  | 0.70 | 0.56-0.88* |  | 0.91 | 0.71-1.16 |  | 0.95 | 0.66-1.36 |
|  |  |  |  |  |  |  |  |  |  |  |  |
| **Child’s age** | | |  |  | |  |  | |  |  | |
| (continuous) | 1.04 | 1.01-1.07* |  | 1.04 | 1.02-1.06* |  | 0.97 | 0.95-0.99* |  | 1.01 | 0.98-1.04 |
| **Child’s ethnicity and race** | | |  |  | |  |  | |  |  | |
| White, non-Hispanic^c^ | *reference* | |  | *reference* | |  | *reference* | |  | *reference* | |
| Hispanic | 0.95 | 0.60-1.50 |  | 0.99 | 0.74-1.33 |  | 1.11 | 0.80-1.56 |  | 1.00 | 0.59-1.70 |
| Black, non-Hispanic | 0.67 | 0.44-1.02 |  | 0.61 | 0.47-0.80* |  | 1.41 | 1.10-1.81* |  | 0.93 | 0.62-1.40 |
| Multiracial or Other | 1.03 | 0.66-1.60 |  | 0.95 | 0.71-1.26 |  | 1.14 | 0.88-1.48 |  | 1.12 | 0.74-1.71 |
| **Parent education** | | |  |  | |  |  | |  |  | |
| Less than high school degree | *reference* | |  | *reference* | |  | *reference* | |  | *reference* | |
| High school degree or GED | 0.92 | 0.41-2.09 |  | 0.95 | 0.52-1.74 |  | 0.81 | 0.46-1.43 |  | 0.31 | 0.13-0.72* |
| Some college or technical school | 0.94 | 0.45-1.97 |  | 0.60 | 0.34-1.07 |  | 0.83 | 0.48-1.42 |  | 0.24 | 0.11-0.53* |
| College degree or higher | 0.76 | 0.36-1.59 |  | 0.44 | 0.25-0.79* |  | 0.59 | 0.34-1.01 |  | 0.22 | 0.10-0.50* |
| **Household language** | | |  |  | |  |  | |  |  | |
| English | *reference* | |  | *reference* | |  | *reference* | |  | *reference* | |
| Non-English | 0.59 | 0.28-1.22 |  | 0.73 | 0.42-1.25 |  | 0.94 | 0.55-1.60 |  | 1.82 | 0.86-3.83 |
| **Insurance type** | | |  |  | |  |  | |  |  | |
| Any private | *reference* | |  | *reference* | |  | *reference* | |  | *reference* | |
| Only public | 1.10 | 0.77-1.56 |  | 1.09 | 0.88-1.34 |  | 1.51 | 1.22-1.86* |  | 1.54 | 1.10-2.16* |
| Uninsured | 1.61 | 0.90-2.86 |  | 0.66 | 0.36-1.18 |  | 1.52 | 0.87-2.68 |  | 1.74 | 0.90-3.36 |
| ^a^aOR = adjusted odds ratio, ^b^CI = confidence interval  ^c^Utilized as reference group because of previous literature demonstrating higher rates of SDM among non-Hispanic white families compared with other racial and ethnic groups.  *Significance level p<0.05  All regressions utilize survey sample weighting for nationally representative inferences | | | | | | | | | | | |

| **Appendix 2: Adjusted Logistic Regressions of Shared Decision-Making (SDM) on Child Health and Healthcare Utilization Outcomes among Children WITHOUT Special Health Care Needs subpopulation** | | | | | | | | | | | |
| --- | --- | --- | --- | --- | --- | --- | --- | --- | --- | --- | --- |
|  | **Forgone Health Care** | |  | **Missed School Days** | |  | **Any ED Visit** | |  | **Multiple ED Visits** | |
|  | **aOR**^a^ | **95% CI**^b^ |  | **aOR** | **95% CI** |  | **aOR** | **95% CI** |  | **aOR** | **95% CI** |
| **Experienced SDM?** | | |  |  | |  |  | |  |  | |
| No | *reference* | |  | *reference* | |  | *reference* | |  | *reference* | |
| Yes | 0.29 | 0.19-0.44* |  | 0.98 | 0.74-1.29 |  | 0.94 | 0.72-1.23 |  | 0.93 | 0.56-1.55 |
|  |  |  |  |  |  |  |  |  |  |  |  |
| **Child’s age** | | |  |  | |  |  | |  |  | |
| (continuous) | 1.05 | 1.02-1.08* |  | 0.98 | 0.95-1.00 |  | 0.97 | 0.95-0.98* |  | 0.95 | 0.92-0.98* |
| **Child’s ethnicity and race** | | |  |  | |  |  | |  |  | |
| White, non-Hispanic^c^ | *reference* | |  | *reference* | |  | *reference* | |  | *reference* | |
| Hispanic | 1.52 | 0.92-2.50 |  | 1.06 | 0.78-1.43 |  | 1.42 | 1.10-1.83* |  | 1.18 | 0.71-1.95 |
| Black, non-Hispanic | 1.69 | 0.89-3.21 |  | 0.49 | 0.34-0.70* |  | 1.16 | 0.87-1.54 |  | 1.82 | 1.04-3.19* |
| Multiracial or Other | 0.83 | 0.50-1.37 |  | 0.92 | 0.66-1.28 |  | 0.89 | 0.68-1.16 |  | 0.72 | 0.38-1.37 |
| **Parent education** | | |  |  | |  |  | |  |  | |
| Less than high school degree | *reference* | |  | *reference* | |  | *reference* | |  | *reference* | |
| High school degree or GED | 0.92 | 0.30-2.80 |  | 1.14 | 0.50-2.60 |  | 0.84 | 0.44-1.64 |  | 1.01 | 0.43-2.37 |
| Some college or technical school | 1.64 | 0.55-4.87 |  | 1.04 | 0.46-2.36 |  | 0.73 | 0.38-1.40 |  | 1.14 | 0.50-2.60 |
| College degree or higher | 0.77 | 0.24-2.50 |  | 0.83 | 0.37-1.86 |  | 0.50 | 0.26-0.95* |  | 0.68 | 0.30-1.52 |
| **Household language** | | |  |  | |  |  | |  |  | |
| English | *reference* | |  | *reference* | |  | *reference* | |  | *reference* | |
| Non-English | 1.47 | 0.81-2.68 |  | 0.46 | 0.27-0.79* |  | 0.66 | 0.44-1.01 |  | 1.05 | 0.56-1.97 |
| **Insurance type** | | |  |  | |  |  | |  |  | |
| Any private | *reference* | |  | *reference* | |  | *reference* | |  | *reference* | |
| Only public | 1.28 | 0.68-2.44 |  | 1.79 | 1.40-2.30 |  | 1.67 | 1.34-2.09* |  | 0.85 | 0.54-1.34 |
| Uninsured | 5.97 | 2.91-12.28* |  | 1.25 | 0.71-2.22 |  | 1.94 | 1.21-3.11* |  | 0.61 | 0.25-1.49 |
| ^a^aOR = adjusted odds ratio, ^b^CI = confidence interval  ^c^Utilized as reference group because of previous literature demonstrating higher rates of SDM among non-Hispanic white families compared with other racial and ethnic groups.  *Significance level p<0.05  All regressions utilize survey sample weighting for nationally representative inferences | | | | | | | | | | | |

| **Appendix 3: Adjusted Logistic Regressions of Shared Decision-Making (SDM) on Child Health and Healthcare Utilization Outcomes with Interaction Term (SDM x Special Health Care Needs Status [CSHCN])** | | | | | | | | | | | |
| --- | --- | --- | --- | --- | --- | --- | --- | --- | --- | --- | --- |
|  | **Forgone Health Care** | |  | **Missed School Days** | |  | **Any ED Visit** | |  | **Multiple ED Visits** | |
|  | **aOR**^a^ | **95% CI**^b^ |  | **aOR** | **95% CI** |  | **aOR** | **95% CI** |  | **aOR** | **95% CI** |
| **Experienced SDM?** | | |  |  | |  |  | |  |  | |
| No | *reference* | |  | *reference* | |  | *reference* | |  | *reference* | |
| Yes | 0.26 | 0.17-0.39* |  | 1.00 | 0.75-1.32 |  | 0.92 | 0.71-1.20 |  | 1.02 | 0.61-1.72 |
| **Child with special health care needs?** |  | |  |  | |  |  | |  |  | |
| No | *reference* | |  | *reference* | |  | *reference* | |  | *reference* | |
| Yes | 2.45 | 1.59-3.78* |  | 2.31 | 1.64-3.24* |  | 1.32 | 0.93-1.86 |  | 2.49 | 1.40-4.42* |
| **Interaction term (SDM x CSHCN)** |  | |  |  | |  |  | |  |  | |
| Yes x Yes | 0.76 | 0.45-1.27 |  | 0.69 | 0.48-0.99* |  | 1.00 | 0.69-1.43 |  | 0.92 | 0.50-1.72 |
|  |  |  |  |  |  |  |  |  |  |  |  |
| **Child’s age** | | |  |  | |  |  | |  |  | |
| (continuous) | 1.04 | 1.02-1.06* |  | 1.01 | 0.99-1.02 |  | 0.97 | 0.96-0.98* |  | 0.98 | 0.96-1.00 |
| **Child’s ethnicity and race** | | |  |  | |  |  | |  |  | |
| White, non-Hispanic^c^ | *reference* | |  | *reference* | |  | *reference* | |  | *reference* | |
| Hispanic | 1.14 | 0.81-1.62 |  | 1.04 | 0.84-1.29 |  | 1.28 | 1.04-1.58* |  | 1.02 | 0.71-1.48 |
| Black, non-Hispanic | 0.94 | 0.63-1.38 |  | 0.57 | 0.46-0.70* |  | 1.28 | 1.06-1.55* |  | 1.21 | 0.86-1.69 |
| Multiracial or Other | 0.95 | 0.68-1.34 |  | 0.92 | 0.74-1.14 |  | 0.99 | 0.82-1.20 |  | 0.94 | 0.66-1.33 |
| **Parent education** | | |  |  | |  |  | |  |  | |
| Less than high school degree | *reference* | |  | *reference* | |  | *reference* | |  | *reference* | |
| High school degree or GED | 0.91 | 0.45-1.83 |  | 1.04 | 0.63-1.70 |  | 0.81 | 0.52-1.26 |  | 0.59 | 0.33-1.04 |
| Some college or technical school | 1.12 | 0.58-2.17 |  | 0.75 | 0.46-1.22 |  | 0.76 | 0.50-1.17 |  | 0.54 | 0.31-0.92* |
| College degree or higher | 0.73 | 0.37-1.44 |  | 0.58 | 0.36-0.94* |  | 0.53 | 0.34-0.81* |  | 0.43 | 0.25-0.74* |
| **Household language** | | |  |  | |  |  | |  |  | |
| English | *reference* | |  | *reference* | |  | *reference* | |  | *reference* | |
| Non-English | 1.06 | 0.64-1.75 |  | 0.55 | 0.38-0.80* |  | 0.76 | 0.54-1.04 |  | 1.27 | 0.77-2.09 |
| **Insurance type** | | |  |  | |  |  | |  |  | |
| Any private | *reference* | |  | *reference* | |  | *reference* | |  | *reference* | |
| Only public | 1.14 | 0.82-1.60 |  | 1.31 | 1.12-1.56* |  | 1.61 | 1.37-1.88* |  | 1.24 | 0.94-1.63 |
| Uninsured | 3.67 | 2.14-6.30* |  | 0.90 | 0.58-1.39 |  | 1.79 | 1.22-2.62* |  | 1.05 | 0.59-1.87 |
| ^a^aOR = adjusted odds ratio, ^b^CI = confidence interval  ^c^Utilized as reference group because of previous literature demonstrating higher rates of SDM among non-Hispanic white families compared with other racial and ethnic groups.  *Significance level p<0.05  All regressions utilize survey sample weighting for nationally representative inferences | | | | | | | | | | | |

| **Appendix 4: Predictive Margins of Interaction Term (Shared Decision-Making [SDM] x Special Health Care Needs Status [CSHCN]) from Adjusted Logistic Regression (Appendix 3)** | | | | | | | | |
| --- | --- | --- | --- | --- | --- | --- | --- | --- |
|  | **Forgone Health Care** | | **Missed School Days** | | **Any ED Visit** | | **Multiple ED Visits** | |
| **SDM x CSHCN** | **Margin** | **95% CI^a^** | **Margin** | **95% CI** | **Margin** | **95% CI** | **Margin** | **95% CI** |
| No x No | 0.13 | 0.09-0.17 | 0.31 | 0.26-0.37 | 0.26 | 0.21-0.30 | 0.18 | 0.11-0.26 |
| No x Yes | 0.26 | 0.21-0.31 | 0.51 | 0.45-0.56 | 0.31 | 0.26-0.36 | 0.36 | 0.28-0.42 |
| Yes x No | 0.04 | 0.03-0.04 | 0.31 | 0.29-0.33 | 0.24 | 0.23-0.26 | 0.19 | 0.16-0.22 |
| Yes x Yes | 0.07 | 0.06-0.08 | 0.42 | 0.40-0.44 | 0.29 | 0.28-0.31 | 0.34 | 0.30-0.38 |
| ^a^CI = confidence interval  All regressions utilize survey sample weighting for nationally representative inferences | | | | | | | | |
